# MicroRNA-146a Regulates Human Foetal Femur Derived Skeletal Stem Cell Differentiation by Down-Regulating SMAD2 and SMAD3

**DOI:** 10.1371/journal.pone.0098063

**Published:** 2014-06-03

**Authors:** Kelvin S. C. Cheung, Nunzia Sposito, Patrick S. Stumpf, David I. Wilson, Tilman Sanchez-Elsner, Richard O. C. Oreffo

**Affiliations:** 1 Bone and Joint Research Group, Institute of Developmental Sciences, Southampton General Hospital, Southampton, United Kingdom; 2 Centre for Human Development, Stem Cells and Regeneration, Human Development and Health, University of Southampton, Southampton, United Kingdom; 3 Clinical and Experimental Sciences, University of Southampton, Southampton, United Kingdom; King Saud University, Saudi Arabia

## Abstract

MicroRNAs (miRs) play a pivotal role in a variety of biological processes including stem cell differentiation and function. Human foetal femur derived skeletal stem cells (SSCs) display enhanced proliferation and multipotential capacity indicating excellent potential as candidates for tissue engineering applications. This study has examined the expression and role of miRs in human foetal femur derived SSC differentiation along chondrogenic and osteogenic lineages. Cells isolated from the epiphyseal region of the foetal femur expressed higher levels of genes associated with chondrogenesis while cells from the foetal femur diaphyseal region expressed higher levels of genes associated with osteogenic differentiation. In addition to the difference in osteogenic and chondrogenic gene expression, epiphyseal and diaphyseal cells displayed distinct miRs expression profiles. miR-146a was found to be expressed by human foetal femur diaphyseal cells at a significantly enhanced level compared to epiphyseal populations and was predicted to target various components of the TGF-β pathway. Examination of miR-146a function in foetal femur cells confirmed regulation of protein translation of SMAD2 and SMAD3, important TGF-β and activin ligands signal transducers following transient overexpression in epiphyseal cells. The down-regulation of SMAD2 and SMAD3 following overexpression of miR-146a resulted in an up-regulation of the osteogenesis related gene RUNX2 and down-regulation of the chondrogenesis related gene SOX9. The current findings indicate miR-146a plays an important role in skeletogenesis through attenuation of SMAD2 and SMAD3 function and provide further insight into the role of miRs in human skeletal stem cell differentiation modulation with implications therein for bone reparation.

## Introduction

Skeletogensis is a multistep process consisting of mesenchymal cell condensation, proliferation, hypertrophic differentiation of chondrocytes, and finally, mineralization of extracellular matrix by osteoblasts [1]–[3]. The process of skeletogensis is orchestrated by various factors including transcription factors [4], micro environmental signals and epigenetic cues [5], [6]. Defects in the regulators of skeletogensis results in skeletal dysplasias, growth failure [2]. A clearer understanding of skeletal stem and bone cell formation and function is critical to inform bone formation strategies and subsequently restore the function of the skeletal system. The cell responsible for bone formation, the osteoblast, is derived from a multipotential marrow stromal stem cell termed the mesenchymal stem cell (undifferentiated multipotent cells of the mesenchyme) which has gained wide acceptance, however this term is non-specific and the term skeletal stem cell (SSC) will be applied to restrict description to stem cells from bone able to generate all skeletal tissues.

MicroRNAs (miRs) are a class of non-protein coding small RNA molecules of 21–25 nucleotides in length. Along with the RNA-induce-silencing complex (RICS), they possess the ability to regulate protein translation by inhibiting their target mRNAs function [7]. There are cumulative evidences to suggest miRs plays an important role in many cellular processes including cell cycle and stem cell differentiation [8], [9]. Various miRs have already been identified to play a role in SSC differentiation, a recent review by Lian *et al* have summarized the effects of 42 miRs on osteoblast differentiation through targeting various cells signaling pathways such as Wnt and TGF-β, transcription factors such as RUNX2 and Osterix and epigenetic machineries such as histone deacetylase 5 (HDAC5) [10]. Data gathered through proteomic approach have demonstrated that a single miR can repress the production of hundreds of proteins, however, the effect of a single miR on protein translation is surprisingly small [11], therefore it can be difficult to determine how a single miR is able to provoke a detectable functional change.

Human foetal femur derived SSC have been shown to contain stromal antigens positive cells with the potential to differentiate down osteogenic, chondrogenic and adipogenic lineages when treated with appropriate culture conditions [12]. Furthermore, foetal femur cell populations have been shown to possess enhanced renewing, differentiation and immunoprivilege potentials, indicating their potential as a cell source for tissue engineering applications [12], [13]. However, cells isolated from the foetal femur comprise a heterogeneous population of cells with differing affinity and capacity for chondrogenic and osteogenic differentiation [14] all of which has served to limit their clinical translation.

A number of growth factors, signaling molecules and transcription factors have been shown to affect skeletal stem cell and osteoprogenitor cell activity including members of the Wnt and TGF-β families [15]–[17]. Furthermore, a number of miRs have been reported to be involved in the regulation of osteogenesis and chondrogenesis through their ability to regulate transcription factors [18]. Thus, miR-140 has been identified as a cartilage specific miR capable of promoting chondrogenic differentiation by increasing the expression of RUNX2, a gene important in chondrocyte hypertrophic differentiation, through down-regulating HDAC4 [19], [20]. More recently, miR-138 was reported by Eskilden and coworkers to be a negative regulator of osteogenic differentiation through inhibition of the expression of Osterix (OSX) via targeting focal adhesion kinase (FAK) [21]. Thus an understanding of the interactions of specific miRs with signaling pathways and growth factors that modulate bone cell function offers new strategies to manipulate and modulate SSC differentiation enhancing our understanding of bone physiology and function critical in any reparative approach.

The current study has examined the expression of miRs within human foetal femur derived diaphyseal and epiphyseal populations. Following identification of select miRs, function was examined using transient overexpression analysis for chondrogenic and osteogenic differentiation. We demonstrate that cells isolated from the epiphyseal regions of the developing foetal femur expressed higher levels of chondrogenic related genes while cells from the diaphyseal region expressed genes associated with osteogenesis. Using RT-qPCR methods, we have confirmed the expression of stromal antigens CD63, ALCAM and putative stem cell marker Nucleostemin by both epiphyseal and diaphyseal cells. MicroRNA microarray analysis confirmed differential miR expression in epiphyseal and diaphyseal populations. Furthermore, putative target and functional analysis demonstrated that miR-146a affected skeletal cell differentiation by down-regulation of SMAD2 and SMAD3 protein translation, genes known to be involved in the activation of chondrogenesis [22]. The current findings indicate an important role of miR-146a, in conjunction with other miRs already described in literature during skeletogenesis and will inform our understanding of bone development and reparation.

## Method

### Ethics Statement

Human foetal tissue was obtained with informed and written consent from women undergoing termination of pregnancy procedure according to guidelines issued by the Polkinghorne Report. Ethical approval was obtained from the Southampton & South West Hampshire Local Research Ethics Committee (LREC 296100).

### Isolation and Culture of Foetal Femur Derived Cells

Foetal femurs samples at 7–9 weeks post conception were isolated from fetuses. Surrounding skeletal muscle and connective tissues were removed from the collected foetal femur samples and separated into epiphysis (region containing densely packed cells) and diaphysis (mid shaft region containing hypertrophic cells) sections by micro-dissection. Femurs were plated into a well of a 6-well-plate (Costar) overnight in α-MEM (Gibco) containing 1 mg/ml collagenase B (Roche). The cell suspension was passed through a 70 µm filter, centrifuged and resuspended in α-MEM supplemented with 10% foetal calf serum (FCS) and 1% penicillin/streptomycin mix (P/S). Cells were maintained in monolayer culture under standard condition until 90% cell confluence was reached before passaging using 1x Trypsin solution (Lonza). All monolayer experiments were conducted on passage two cells. Foetal age was determined by measuring foetal foot length and samples classified as weeks post conception.

### Osteogenic and Chondrogenic Conditions

Cells were passaged into six-well plates for osteogenic and chondrogenic differentiation. Control cultures were refreshed with basal medium (α-MEM supplemented 10% FCS) every 48 hours. For osteogenic culture, cells were cultured using medium supplemented with 10% FCS, 10 nM of dexamethasone and 100 µM of ascorbic acid 2-phosphate. For Chondrogenic culture: α-MEM containing 5 ng/ml TGF-β3, 10 nM dexamethasone, 100 µM ascorbate, and 10 µl/ml of 100x ITS liquid media (Sigma I3146) was used.

### Alcian Blue/Sirius Red Staining

Whole foetal femurs were fixed with 4% PFA-PBS overnight at 4°C and processed through graded ethanol. Samples were immersed in 50% chloroform/ethanol and 100% chloroform. Samples were placed in paraffin wax at 60°C for 30 minutes to enable full penetration of paraffin wax. Thereafter, tissues were embedded in paraffin wax blocks for sectioning. Sections were cut on a Microm 330 at 6 µm thick and the cut sections were transferred onto pre-heated glass slide. Prior to staining, the samples were processed through histoclear solution twice to remove paraffin wax and rehydrated using reverse graded methanol solutions (100%, 100%, 90% and 50%). To allow visualization of the cell nucleus, Weigert’s haematoxylin solution was prepared and added to rehydrated samples for 10 minutes. Excess staining solution was removed with water and acid alcohol. To stain for proteoglycans, samples were immersed in 0.5% Alcian blue 8GX solution. Samples were placed in 1% molybdophosphoric acid followed by incubation in 0.1% Sirius red F3B solution to stain for collagen. Sections were rinsed thoroughly with water and dehydrated in reverse graded methanol back into histoclear before mounting in dibutyl phthalate xylene (DPX).

### Image Capture and Analysis

Sample images were captured using a Zeiss Axiovert 200 inverted microscope and Zeiss Axiovision software version 4.7. Light microscopy images were taken using an Axiocam HR camera and fluorescent images were captured using Axiocam MR.

### RNA Extraction and RT-PCR Analysis

RNA extraction was performed using mirVana RNA Isolation System Kit (Life Technologies) according to the manufacturer’s protocol. Briefly, cultured samples were placed on ice and washed twice in PBS. Lysis buffer was used to release RNA and a miR homogenizing agent was added, followed by acid phenol-chloroform. The resultant mixture was centrifuged to allow phase separation. The aqueous phase was removed and added to ethanol prior to elution through a spin column. The column was washed three times with supplied buffer solutions and RNA eluted in RNase free water.

For cDNA synthesis, SuperScript VILO cDNA Synthesis Kit (Life Technologies) was used. RNA was combined with 5X VILO reaction mix and 1 µl of 10X SuperScript enzyme and incubated at 25°C for 10 minutes followed by 42°C for 2 hours. The reaction was terminated by incubation at 85°C for 5 minutes. 40 µl of water was then added to the cDNA sample to give a 1∶4 dilution and stored at −20°C or used immediately for quantitative RT-PCR analysis. Quantitative RT-PCR was performed using SYBR-Green PCR master mix (Life Technologies): 10 µl of SYBR-Green master mix; 5 µl of up H_2_O; 2 µl of forward and reverse primers for the gene of interest (Table 1) and 1 µl of cDNA sample. The final mixture (20 µl) was added to each well of a 96-well-plate, analyzed using an Applied Biosystem (Life Technologies), 7500 Real Time PCR system. Data was analyzed using the Applied Biosystem 7500 System SDS Software, version 2.0.5. Ct value for each sample was normalized to β-Actin, an endogenous housekeeping gene, and fold expression levels for each target gene were calculated using the delta-delta Ct (Cycle threshold) method.

**Table 1:** Forward and reverse primers sequence used for RT-qPCR.

Gene	Primer sequences	Amplicon size
***Human β- Actin***	F: 5′ ggc atc ctc acc ctg aag ta 3′	82 bp
	R: 5′ agg tgt ggt gcc aga ttt tc 3′	
***Human Runx-2***	F: 5′ gta gat gga cct cgg gaa cc 3′	78 bp
	R: 5′ gag gcg gtc aga gaa caa ac 3′	
***Human ALP***	F: 5′ gga act cct gac cct tga cc 3′	86 bp
	R: 5′ tcc tgt tca gct cgt act gc 3′	
***Human Col1a1***	F: 5′ gag tgc tgt ccc gtc tgc 3′	52 bp
	R: 5′ ttt ctt ggt cgg tgg gtg 3′	
***Human Type X Collagen***	F: 5′ ccc act acc caa cac caa ga 3′	95 bp
	R: 5′ gtg gac cag gag tac ctt gc 3′	
***Human Osteonectin***	F: 5′ gag gaa acc gaa gag gag g 3′	95 bp
	R: 5′ ggg gtg ttg ttc tca tcc ag 3′	
***Human SMAD3***	F: 5′ tga atc cct acc act acc aga g 3′	117 bp
	R: 5′ gga tgg aat ggc tgt agt cg 3′	
***Human SMAD 2***	F: 5′ gat cct aac aga act tcc gcc 3′	146 bp
	R: 5′ cac ttg ttt ctc cat ctt cac tg 3′	
***Human Sox9***	F: 5′ ccc ttc aac ctc cca cac ta 3′	74 bp
	R: 5′ tgg tgg tcg gtg tag tcg ta 3′	
***Human Col2a1***	F: 5′ cct ggt ccc cct ggt ctt gg 3′	58 bp
	R: 5′ cat caa atc ctc cag cca tc 3′	
***Human Nucleostemin***	F: 5′ ggg aag ata acc aag cgt gtg 3′	98 bp
	R: 5′ cct cca aga agt ttc caa agg 3′	
***Human CD63***	F: 5′ gcc ctt gga att gct ttt gtc g 3′	87 bp
	R: 5′ cat cac ctc gta gcc act tct g 3′	
***Human ALCAM***	F: 5′ acg atg agg cag acg aga taa gt 3′	96 bp
	R: 5′ cag caa gga gga gac caa caa c 3′	

### MicroRNA Microarray Analysis

Applied Biosystems TaqMan Low Density Array system was used to perform RT-PCR miR arrays, following the manufacturer’s protocol. In brief, 400 ng of total RNA was used to generate cDNA using MegaplexRT Human Pool A (Life Technologies). 6 µl of cDNA was then combined with 450 µl of 2x TaqMan PCR Master mix (Life Technologies) and 444 µl of water before loading into each micro array card. An Applied Biosystems 7900 thermocycler was used to run the array. The raw data generated were analysed using Sequence Detection System v2.4 Enterprise edition (Applied Biosystems). Further data analysis and heat maps were created in R (v 2.15.3). Raw expression values were pre-processed using the R package qpcrNorm [23]. Heat maps were generated from quantile normalized, z-transformed Ct values, ordered by ascending ddCt of epiphysis versus diaphysis. A subset of all expressed miRss was selected for a heat map based on statistical evidence for differential expression according to a permissive t-test (α = 0.1).

### MicroRNA Expression Analysis

MicroRNA expression was examined using TaqMan MicroRNA Assays (Life Technology). Each assay comprised two primers, one for cDNA synthesis and one for TaqMan RT-PCR. cDNA specific to each assay-specific miR was generated using TaqMan MicroRNA Reverse Transcription Kit (Life Technologies) from total RNA using a modified manufacturer’s protocol. In brief, a reaction mixture containing 50 mM dNTPs, 25 units of Mutiscribe RT enzyme, 0.75 µl 10x Buffer, 1.88 units of RNase inhibitor, 3.58 µl water, 1.5 µl of primer and 10 ng of total RNA was prepared. The reaction mixture was mixed by pipetting and incubated at 16°C for 30 minutes followed by 42°C for 30 minutes. The reaction was terminated following incubation for 5 minutes at 85°C. The cDNA sample was stored at −20°C if not used immediately for qPCR analysis.

For the qPCR reaction, TaqMan Universal PCR Master Mix with No AmpErase UNG (Life Technologies) was utilised. A reaction mixture containing 5 µl TaqMan master mix, 3.335 µl of up H_2_O, 0.5 µl of qPCR primer and 0.7 µl of cDNA was prepared. The mixture was transferred into the well of a 96-well plate and loaded into the Applied Biosystem (Life Technology, USA), 7500 Real Time PCR system for assay. All reactions were performed in duplicate and included a negative control lacking cDNA. The data was analyzed using the Applied Biosystem 7500 System SDS Software, version 2.0.5 program. Ct value for each sample was normalized to MammU6, an endogenous control for miR and fold expression levels for each target gene was calculated using the delta-delta Ct (Cycle threshold) method.

### MicroRNA Transient Overexpression

To assess the effect of miR-146a on cultured cells, transient over-expression of miR-146a was achieved using a miR-146a mimic and DharmaFECT reagent (Dharmacon). Briefly, epiphyseal cells were expanded in culture until 80% confluent. Cells were trypsinised and plated to each well of a 24-well plate in α-MEM supplemented with 10% FCS and 1% P/S and cultured for 24 hours. On transfection, cells were washed with PBS and transfection media containing DharamFECT reagent and 100 ηm of miR-146a mimic was added to cultured cells. For the control group, cells were transfected with a scrambled miR mimic (Dharmacon) under the same condition. RNA was then extracted for RT-PCR analysis 48 hours and protein was obtained after 72 hours post transfection for western blot analysis.

### Western Blot Analysis

Cells were lysed with RIPA buffer (750 mM NaCl, 5% IgePal CA-630, 2.5% DOC, 0.5% SDS, 250 mM Tris (pH 8.0)) supplemented with a protease inhibitor cocktail (1∶100; Sigma). Cell lysates were centrifugation at 13000 rpm for 20 minutes at 4°C, and the supernatant collected. Protein concentration was determined using Pierce BCA protein Assay Kit (Pierce). 20 µg of each samples combined with DTT (Cell Signalling) and SDA sample buffer (BioLabs) were analysed by SDS gel electrophoresis using Any kD precast gels (Mini-PROTEAN TGX; Bio-rad) and transferred onto a polyvinylidene fluoride (PVDF) immobilon-FL transfer membrane (Millipore). Immunoblots were probed with SMAD3 and SMAD2 antibody (1∶1000; Cellsignalling), then with anti-β-Actin-Peroxidase (1∶10000; Sigma). IRDye 800CW goat anti-rabbit (LI-COR Biosciences) and IRDye 800CW rabbit anti-HRP (LI-COR Biosciences) were used as secondary fluorescent antibodies respectively. The anti-β-actin-Peroxidase was used as loading control to which protein expression across the membrane was normalized. The blots were scanned individually on an Odyssey Infrared Imaging System (LI-COR Biosciences), and densitometry analyses were run calculating the signal intensity ratio between samples and loading control. Image Studio software (LI-COR, Biosciences) was employed to obtain the signal intensity values which were transformed into percentages of intensity for the statistical analysis.

### Statistics

Statistical analysis was carried out using Mann-Whitney test or One-way Analysis of Variance (ANOVA) with Tukey-Kramer multiple comparison post-test using statistics software package Prism version 6 (GraphPad software). Values were expressed as mean ± standard deviation. All experiments were performed using at least three separate foetal samples unless otherwise stated. Values for p≤0.05 were considered statistically significance.

## Results

### Isolation of Osteogenic Diaphyseal and Chondrogenic Epiphyseal Foetal Femur Cells Population

**Figure 1:**
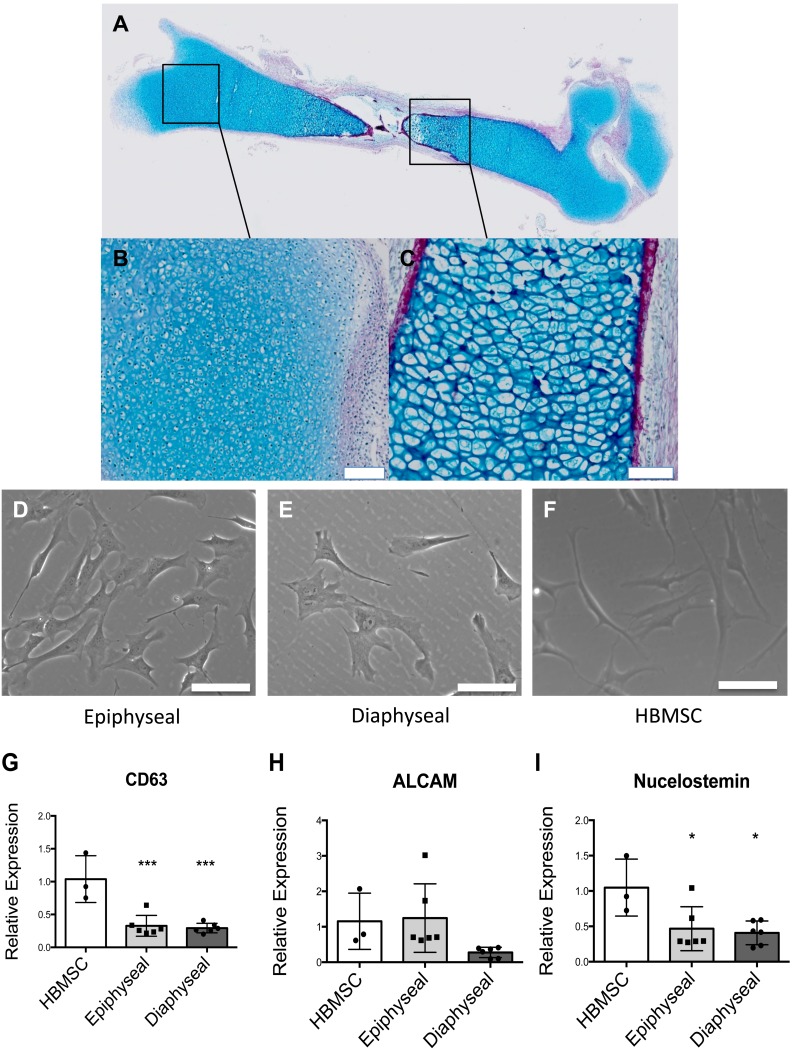
Morphology of foetal femur derived cells in whole femur and post monolayer culture with quantitative expression of stromal antigens and putative stem cell marker by HBMSC, foetal femur epiphyseal and diaphyseal cells. Foetal femur comprised a proteoglycan anlage (Blue) with a bone collar marked by deposition of collagen (Red) (A). Epiphyseal region contained proliferating chondrocytes (B) while diaphyseal section, comprised hypertrophic chondrocytes (C). Epiphyseal cells (D) and diaphyseal cells (E) adopt similar morphology to HBMSC (F) upon monolayer culture. Expression of CD63 (G), ALCAM/CD166 (H) and Nucleostemin (I) by foetal femur epiphyseal cells and diaphyseal cells was confirmed by RT-qPCR and expression levels compared to human bone marrow stromal cells (set to have expression of one). Results expressed as mean ± SD and n = 3. ***P<0.001 and *P<0.1 calculated using ANOVA. Scale bar = 100 µm.

Foetal femurs were observed to comprise primarily of a cartilage anlage with a developing bone collar marked by collagen deposition, as evidenced by Alcian blue and Sirus red staining (Figure 1A). Within the epiphyseal regions, cells were observed to be smaller and densely packed (Figure 1B), while cells from the diaphyseal region displayed a hypertrophic phenotype (Figure 1C). Following monolayer culture, both epiphyseal and diaphyseal derived cell populations formed distinct cell colonies displaying a fibroblastic morphology similar to the human bone marrow stromal cells (Figure 1D–F). RT-qPCR confirmed expression of stromal antigens CD63 and ALCAM (Figure 1G–H) in epiphyseal and diaphyseal cells although at a lower level in comparison to human bone marrow stromal cells (HBMSC) (Figure 1G). Epiphyseal cells expressed ALCAM at a similar level to HBMSC while diaphyseal cells appeared to express lower levels of ALCAM (Figure 1H). Nucleostemin, a putative stem cell marker, was found to be expressed by both epiphyseal and diaphyseal cells (Figure 1I). The expression of CD63, ALCAM and Nucleostemin by epiphyseal and diaphyseal cells in monolayer culture, suggest these cell populations retain mesenchymal progenitor cell-like characteristics. We have previously published on the potential of foetal femur derived cells to differentiated along the stromal osteogenic, chondrogenic and adipogenic lineages [12] and this was not re-examined here.

### Epiphyseal and Diaphyseal Cells Display Distinct Osteogenic and Chondrogenic Differentiation Potential

**Figure 2:**
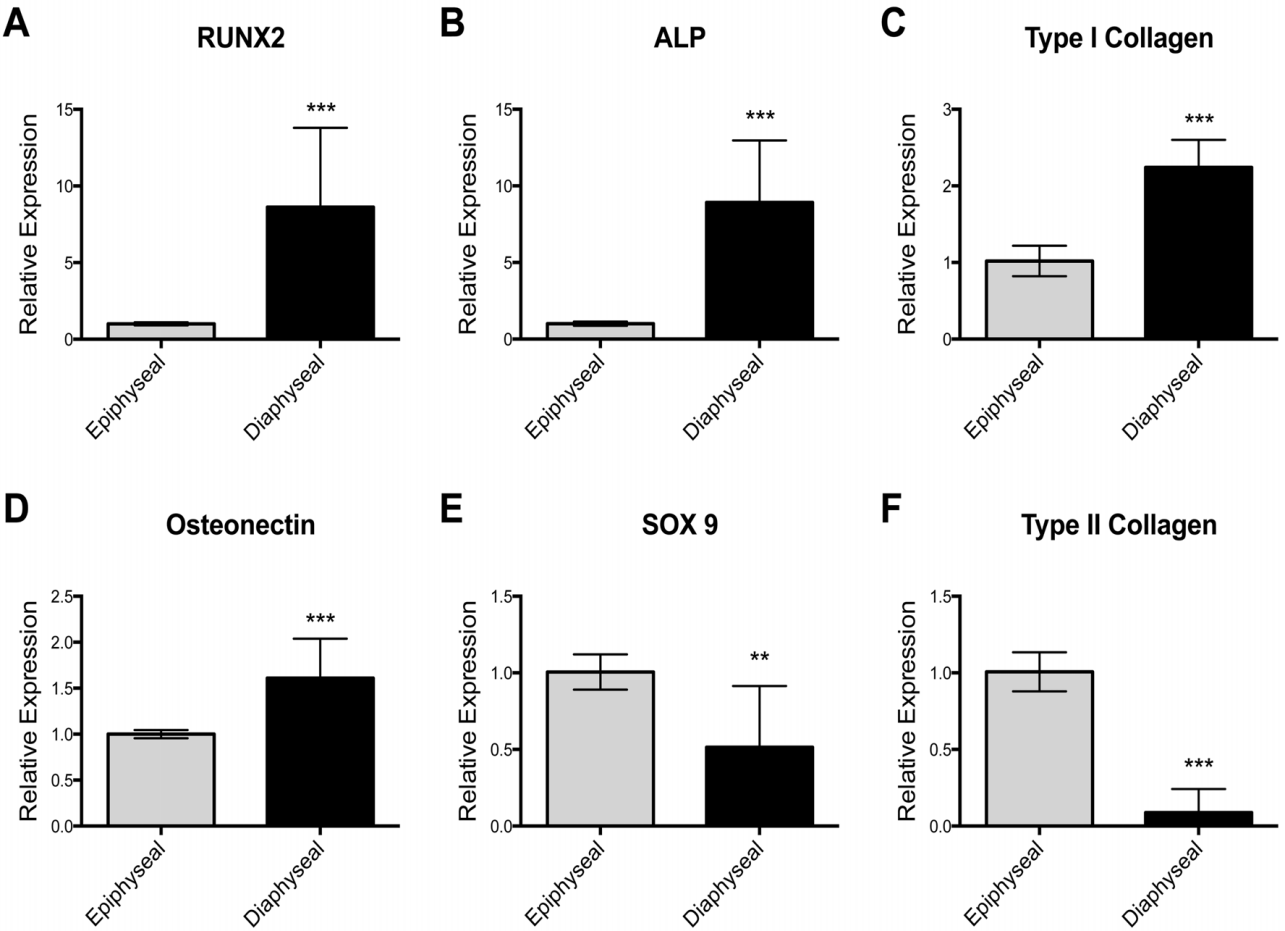
Relative expression (RT-qPCR) of osteogenic and chondrogenic marker genes in epiphyseal and diaphyseal cell population following monolayer culture. Cells extracted from the diaphyseal region expressed higher levels of osteogenic marker genes; *RUNX2*, *ALP*, *Type I collagen* and *Osteonectin* (A–D) while epiphyseal cells expressed higher level of genes associated with chondrogenesis; *SOX9* and *Type II collagen* (E–F). Relative expression was normalized to β-Actin with epiphyseal cell populations set to have an expression of one. Data are represented as an average of three independent patient samples and error bars represent standard deviation. **P<0.01; ***P<0.001.

**Figure 3:**
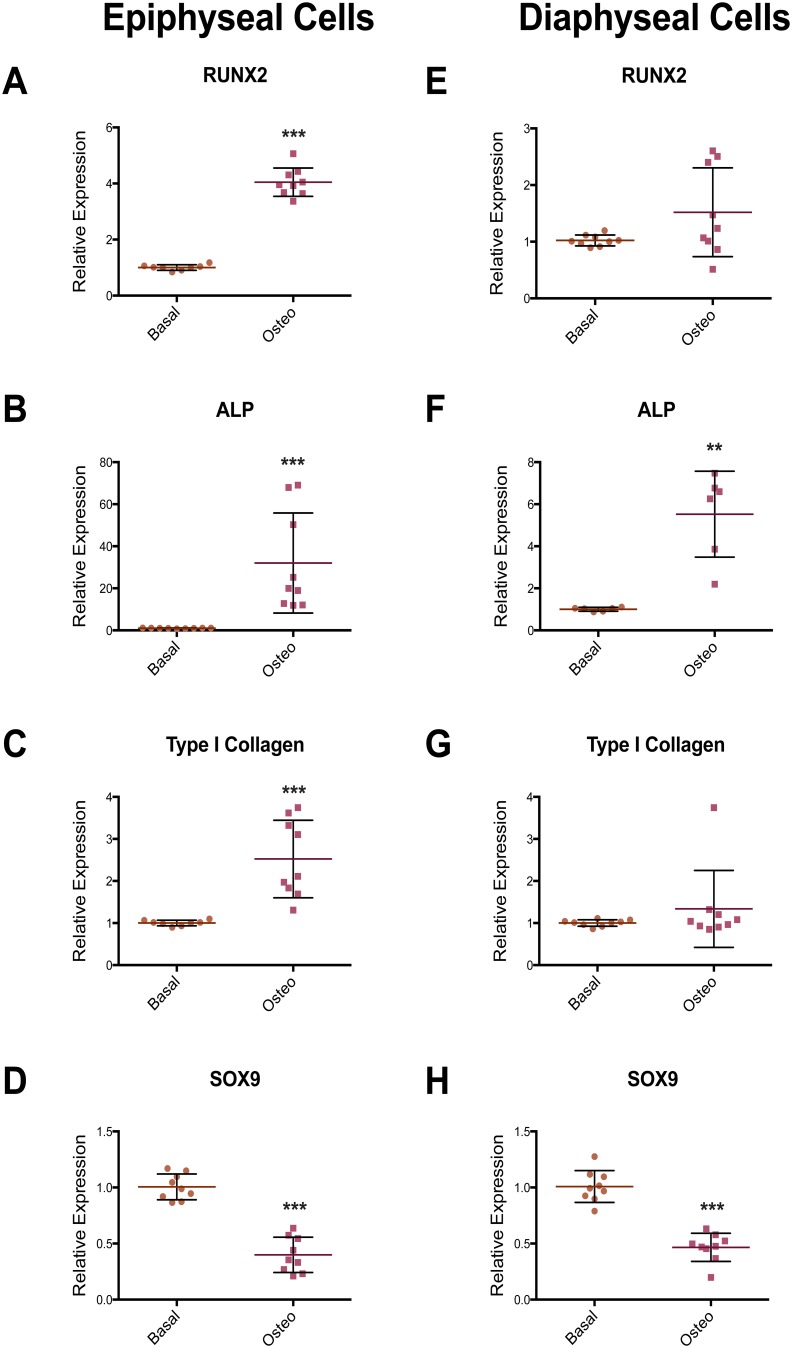
RT-PCR results showing the effect of osteogenic media on osteogenic and chondrogenic gene expression in epiphyseal and diaphyseal cell populations. Epiphyseal cells, in osteogenic media, expressed higher levels of RUNX2, ALP and Type I collagen (A–C) whilst in diaphyseal cell populations, only an increase in ALP expression was recorded (E–G). In epiphyseal and diaphyseal cells, the expression of the chondrogenic transcription factor, SOX9, was reduced in osteogenic media (D and H). Relative expression was normalized to β-Actin with basal condition set to an expression of one. Data represent an average of three independent patient samples and error bars represent standard deviation. **P<0.005; ***P<0.001 calculated using Mann-Whitney test.

Epiphyseal and diaphyseal cells were isolated from three unrelated patient samples. Following 7 days in monolayer culture under basal condition, RNA was extracted from each individual sample for gene expression analysis. Using RT-qPCR, diaphyseal cells were shown to express higher levels of genes associated with the osteoblast phenotype, namely RUNX2, ALP, Type I Collagen and Osteonectin (Figure 2A–D). Epiphyseal cells expressed increased level of genes associated with the chondrocyte phenotype, namely SOX9 and type II collagen (Figure 2E–F). Following culture in osteogenic conditions, epiphyseal and diaphyseal cells expressed higher levels of ALP mRNA (Figure 3B and F), while, RUNX2 and Type I Collagen expression were only observed to be increased in the epiphyseal cell population (Figure 3A and C), suggesting enhanced osteogenic modulation of epiphyseal cell populations by osteogenic media. In epiphyseal and diaphyseal cells, SOX9, a gene associated with chondrocyte differentiation, was reduced following osteogenic media supplementation (Figure 3D and H).

### Epiphyseal and Diaphyseal Cells Express Distinct MicroRNAs

**Figure 4:**
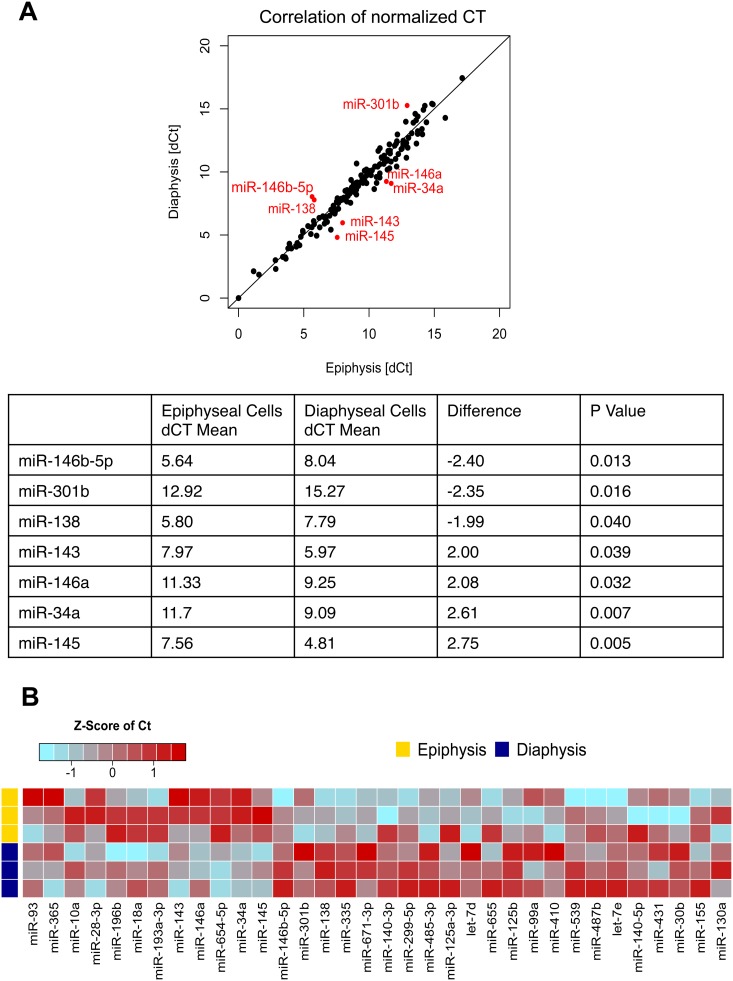
MicroRNA expression profile of epiphyseal and diaphyseal cells. CT values of each miR was normalized to MammU6 (dCT) and plotted as an XY scattered chart displaying the correlation of miR expression by epiphyseal and diaphyseal cells. An unpaired t-test revealed 7 miRs with significant differences in expression: miR-146a-5p, miR-301b and miR-138 displayed higher expression in epiphyseal cells while miR-143, miR-146a, miR-34a and miR-145 displayed higher expression levels in diaphyseal cells (A). A heat map of normalized CT values was generated to show the difference in miR expression between epiphyseal and diaphyseal cell populations (B). The Heat map represents z-transformed expression, blue represents higher level of expression and red represents lower level of expression.

**Figure 5:**
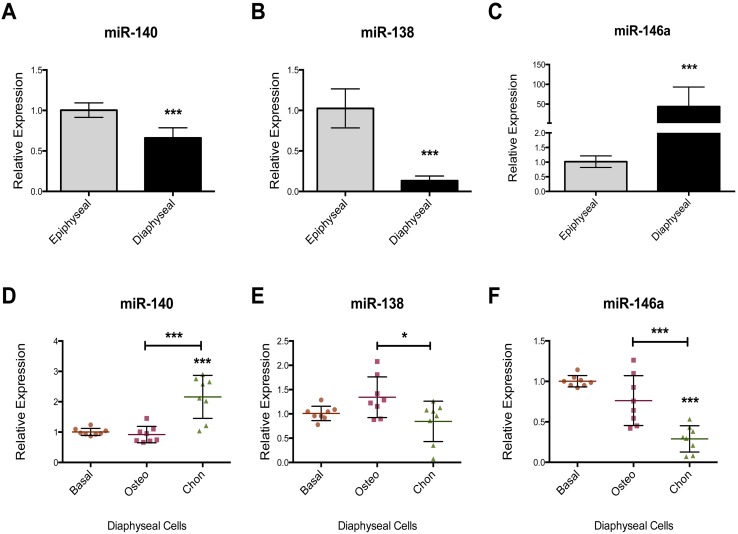
Relative expression (RT-qPCR) of miR-140, miR-138 and miR-146a in epiphyseal and diaphyseal cell populations (A–C) and the effect of differentiation media on expression in diaphyseal cells (D–F). miR-140 was expressed at a higher level in epiphyseal cells (A). miR-138, known to have anti-osteogenic effects was found to have a lower level of expression in diaphyseal cells (B). miR-146a, identified in our microarray, was validated and shown to display a markedly increased expression level in the diaphyseal cell populations compared to epiphyseal cells (C). The use of osteogenic media did not affect the expression levels of miR-140, miR-138 and miR-146a compared to culture in basal conditions (D–F). Chondrogenic culture conditions resulted in increased expression of miR-140 while miR-146a expression was suppressed (D–F). Relative expression was normalized to MAMM-U6 with epiphyseal cells (A–C) and basal condition (D–F) set to an expression of one. Data represent an average of three independent patient samples and error bars represent standard deviation. *P<0.05; **P<0.005; ***P<0.001 calculated using Mann-Whitney (A–C) and ANOVA test (D–F).

RT-qPCR miR array was used to examine the miR expression profile in the chondrogenic epiphyseal cells and osteogenic diaphyseal cells in three unrelated foetal femur samples. The miR array expression analysis indicated 155 out of the 377 detectable miRs were expressed in the samples. Of these, 67 miRs were found have a difference in expression of greater than 1.5 fold between epiphyseal and diaphyseal cells with 12 miRs expressed at an elevated level in epiphyseal cells and 55 miRs in the diaphyseal cell population. Only 7 miRs were identified with a statistically significant difference in expression between epiphyseal and diaphyseal cells (Figure 4A), namely: miR-146b-5p, miR-301b and miR-138 (higher expression in epiphyseal cells) and miR-143, miR-145, miR-146a and miR-34a (increased expression in diaphyseal cells). A heat map was employed to demonstrate the difference in selected miRs expression between epiphyseal and diaphyseal cells (Figure 4B). miR-146a was found to be expressed at a higher level in the diaphyseal cell population and was selected for revalidation using an individual TaqMan RT-qPCR assay. miR-138 and miR-140, have previously been reported to have anti-osteogenic and pro-chondrogenic properties respectively [21], [24] and were found to display a higher expression in epiphyseal cell populations and thus selected for revalidation using individual TaqMan RT-qPCR assay to assess the consistency of data of the current study compared to current literature. In addition, the effects of osteogenic and chondrogenic media on the expression of miR-140, miR-138 and miR-146a were examined. Individual TaqMan assays confirmed the expression of the cartilage specific miR-140 and the anti-osteogenic miR-138 was higher in the epiphyseal cell populations (Figure 5A and B). miR-146a displayed a 50-fold increase in expression in diaphyseal cells relative to epiphyseal cells (Figure 5C). Culture in osteogenic media failed to modulate the expression of miR-138, miR-140 and miR-146a (Figure 5D and E). Critically, the expression of miR-146a was markedly reduced in the presence of chondrogenic media (Figure 5F).

### MicroRNA-146a Target Analysis

**Figure 6:**
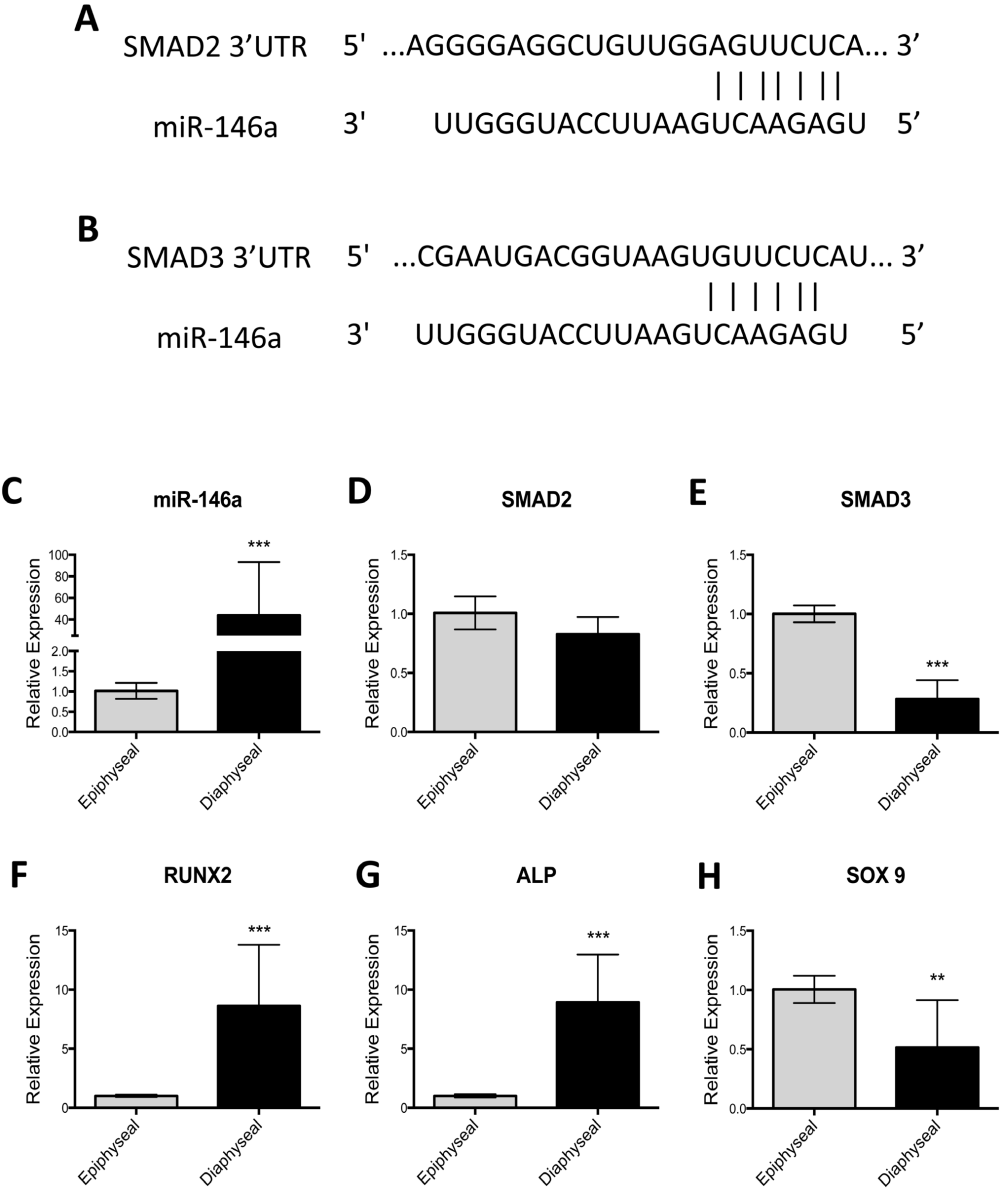
Predicted binding site for miR-146a in SMAD2 and SMAD3 mRNA 3′UTR and the relationship between expression of miR-146a, SMAD2, SMAD3 and Type X Collagen expression in epiphyseal and diaphyseal cell populations. SMAD2 and SMAD3 mRNA 3′UTR contains a binding site for miR predicted by TargetScan (A–B). Increased expression of miR-146a in diaphyseal cell populations was correlated with reduced expression in SMAD3 mRNA levels but not SMAD2 mRNA levels (D–E). Increased expression of miR-146a in diaphyseal cells was coupled with an increase in expression of RUNX2 and ALP (F–G). Reduced expression of SOX9 was observed when miR-146a was highly expressed. Relative expression was normalized to MAMM-U6 (C) and β-Actin (D–H). Data represents an average of three independent patient samples and error bars represent standard deviation. *P<0.05, **P<0.01; ***P<0.001 calculated using Mann-Whitney test.

A number of the miRs identified by the microarray analysis have already been described to be involved in the differentiation of SSC, namely; miR-146b-5p [25], [26], miR-138 [7], [21], miR-143 [8], [9], [27] and miR-145 [10], [28], [29]. However, the effects of miR-146a on bone cell function are unknown. Furthermore, through putative target analysis using Targetscan [30], miR-146a appeared to target various key regulators the TGF-β ligand specific pathway; SMAD2, SMAD3 and SMAD4. SMAD2 and SMAD3 were chosen for validation as they are known intracellular transducers of TGF-β and activin ligands and are thought to play a critical role in chondrogenic differentiation [11], [22] whilst SMAD4 was omitted in the analysis as the effect of miR-146a on SMAD4 function have already been described [12], [31]. The putative binding site of miR-146a to SMAD2 and SMAD3 3′UTR is shown in figure 6A and 6B respectively. To determine whether a correlation existed between miR-146a expression and SMAD2/SMAD3 expression in epiphyseal and diaphyseal cell populations, RT-qPCR expression was undertaken. The increased expression of miR-146a observed in diaphyseal cells was associated with decreased expression of SMAD3 mRNA level however; no correlation to the expression of SMAD2 was observed (Figure 6C–E). Furthermore, enhanced expression of miR-146a was associated with an increased expression of RUNX2 and ALP (6F–G). Critically, decreased expression of SOX9 was observed when miR-146a was highly expressed (Figure 6H).

### MicroRNA-146a Regulates Skeletal Stem Cell Differentiation by Down-regulating SMAD2 and SMAD3 Protein Translation

**Figure 7:**
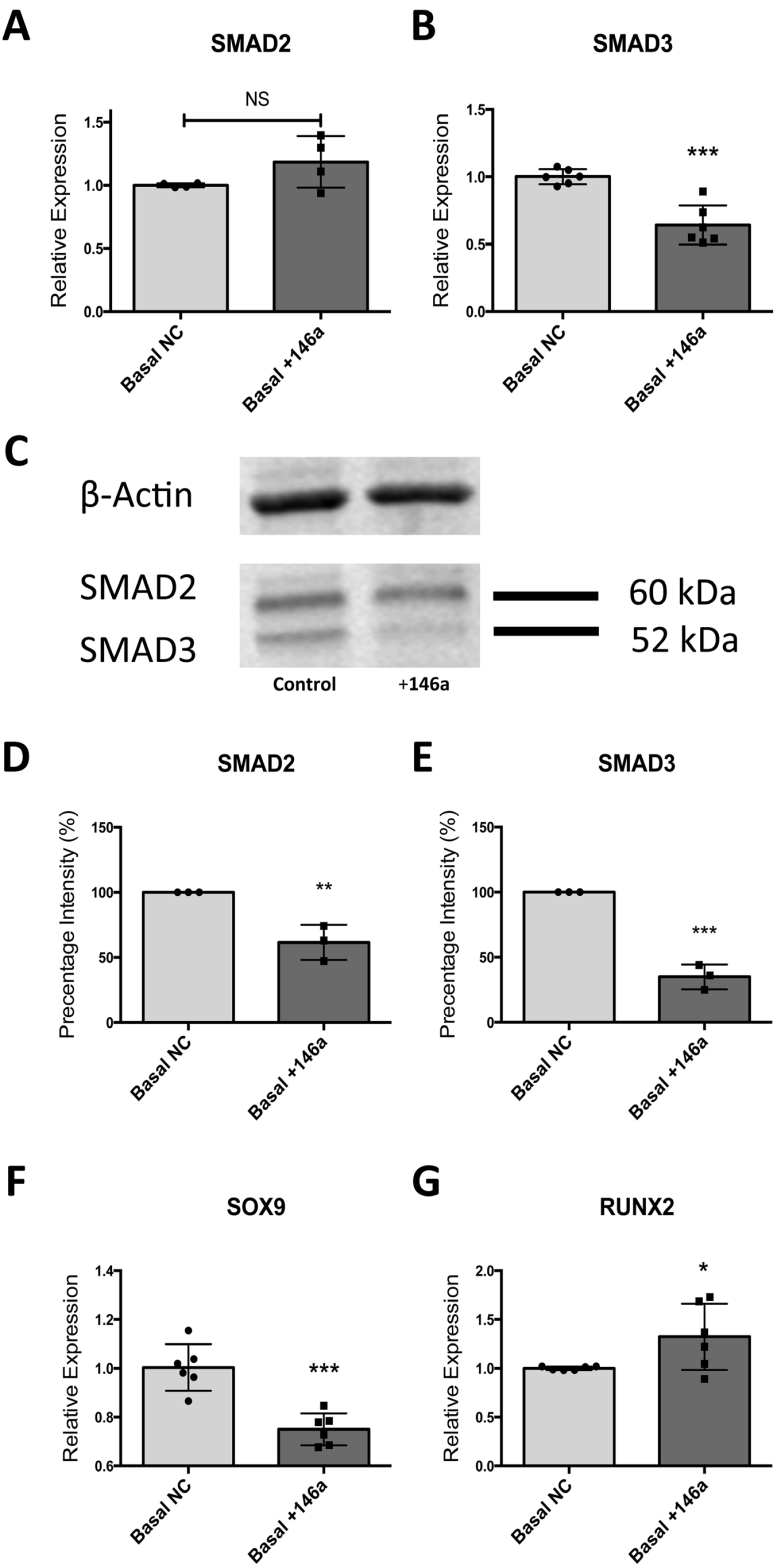
Effect of miR-146a overexpression on SMAD2/SMAD3 mRNA expression and protein translation and in osteogenic related gene expression. Overexpression of miR-146a resulted in a reduction of SMAD3 mRNA expression while SMAD2 mRNA levels remained unchanged (A–B). Protein level of both SMAD2 and SMAD3 were significantly reduced by overexpression of miR-146a as evidenced by western blot analysis (C) with densitometry band intensity quantification (D–E). miR-146a overexpression resulted in increased RUNX2 expression and a reduction in SOX9 expression (F–G). Relative expression analysis by RT-qPCR was normalized to β-Actin. Densitometry data presented as percentage of band intensity compared to control group (100%). Data are represented as an average of three independent patient samples and error bar represents ± SD. *P<0.05, **P<0.01; ***P<0.001 calculated using Mann-Whitney test.

To validate the effect of miR-146a on SMAD2 and SMAD3 protein translation, miR-146a was transiently overexpressed in epiphyseal cells from three independent patient samples using a miR-146a mimic. RNA was extracted 48 hours post transfection and protein was harvested after 72 hours. These time points were chosen after testing the transfection agents used in a series of optimization experiments (data not shown). 48 hours post miR-146a over-expression; down-regulation of SMAD3 mRNA was observed however, SMAD2 mRNA levels remained unchanged (Figure 7A–B). Western Blot analysis showed a reduction in both SMAD2 and SMAD3 protein translation in the presence of miR-146a overexpression. Quantitative analysis using western blots demonstrated miR-146a significantly reduced SMAD3 protein expression over SMAD2 expression (65% and 35% respectively) (Figure 7D–E). The down-regulation in SMAD2 and SMAD3 was coupled with a down-regulation of SOX9 expression and up-regulation of RUNX2 mRNA expression suggesting miR-146a has a positive effect on osteogenesis (Figure 7F–G).

### MicroRNA-146a/TGF-β Feedback Mechanism Regulates Chondrocyte Hypertrophic Differentiation

**Figure 8:**
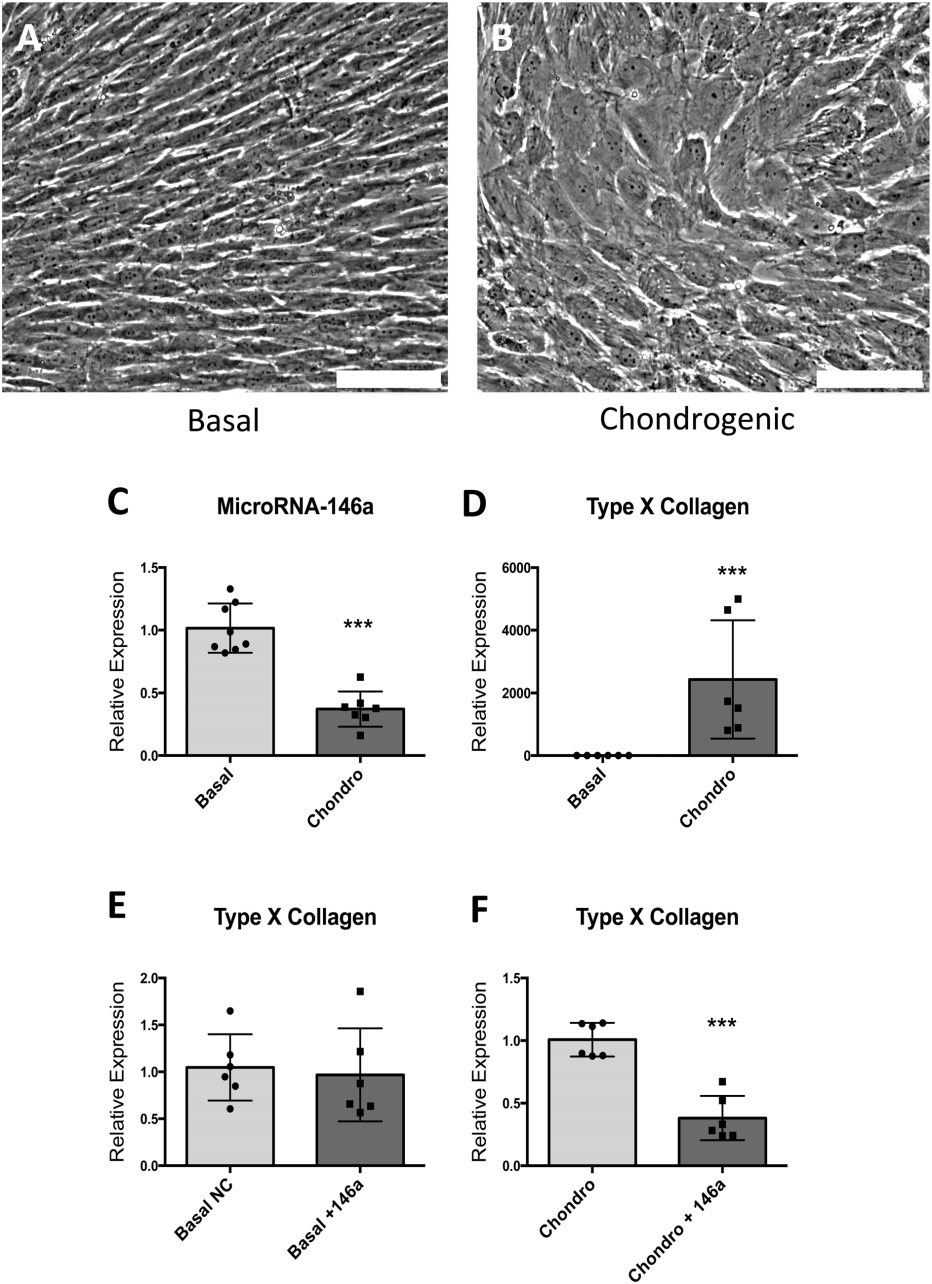
The effect of chondrogenic media containing 4/ml of TGF-β3 on morphology of cells in monolayer culture and the expression of miR-146a and Type X Collagen mRNA. TGF-β3 addition (chondrogenic media) culminated in cells with a distinct hypertrophic phenotype (A–B). Expression of miR-146a was reduced following culture under chondrogenic conditions (C) and a significant up-regulation of Type X collagen was observed (D). Under basal condition, miR-146a expression did not affect the expression of Type X Collagen (E). Under chondrogenic condition, miR-146a over-expression reduced the stimulatory effect of chondrogenic media on type X collagen (F). * = P<0.05, *** = P<0.001. Scale bars represent 100 µm.

Human foetal cells extracted from the epiphyseal layers undergo hypertrophic differentiation in the presence of chondrogenic media containing TGF-β3 (Figure 8A–B). *In vitro* stimulation of epiphyseal cells with chondrogenic media containing TGF- β3 resulted in down-regulation of miR-146a (figure 8C) and a substantial up-regulation of Type X collagen mRNA expression (Figure 8D). To further validate the effect of miR-146a as a negative regulator of TGF-β ligand dependent signaling pathway through down-regulating protein translation of SMAD2 and SMAD3, the effect of TGF-β3 on Type X Collagen in the presence of miR-146a overexpression in basal and chondrogenic conditions was compared. Under basal condition, overexpression of miR-146a had no effect on the expression of Type X Collagen (Figure 8E); however, in the presence of miR-146a overexpression, up-regulation of Type X Collagen by TGF-β3 was reduced by 60%. These results suggesting the effects of miR-146a on the TGF-β pathway are dependent on the presence TGF-β3 ligands (Figure 8F).

## Discussion

MicroRNAs have recently been demonstrated as important regulators of a variety of biological processes [12], [13], [32] including cell cycle, oncogenesis and stem cell differentiation [9], [14], [33], [34]. Using TaqMan RT-qPCR MicroRNA array, epiphyseal and diaphyseal cells were found to express a different repertoire of miRs. Microarray data are known to be relatively noisy [15]–[17], [35] and together with the accepted variation inherent to primary cells samples, any statistical analysis of microarray data can prove challenging. miR-146a was identified to be expressed at a higher level in diaphyseal cells compared to epiphyseal cells, suggesting a role of miR-146a in osteogenic differentiation. Using Targetscan release 6, potential mRNA targets of miR-146a were identified [30]. Various components of the TGF-β pathway; namely SMAD2, SMAD3, SMAD4, TGFB-induced factor homeobox 1 (TGIF1), BMP and activin membrane-bound inhibitor homolog (BAMBI) and activin A receptor type IC/I/Type II-like 1 (ACVR1C/ACVR1B/ACVRL1) were highlighted as potential targets of miR-146a, advocating the function of this miR in diaphyseal cells maybe mediated via attenuation of the TGF-β pathway [30]. miR-146 has previously been identified to modulate myofibroblast trans-differentiation during TGF-β1 induction by targeting SMAD4 [18], [36] and miR-146 may also be an important regulator during the inflammatory state of osteoarthritis, as IL-1β induced production of TNF-α, a pro-inflammatory cytokine known to play a role in osteoarthritis, was significantly reduced by miR-146 overexpression [19], [20], [37]. Furthermore, overexpression of miR-146a has been shown to protect the human bronchial epithelial from apoptosis and to promote cell proliferation through up-regulation of Bcl-XL and STAT3 phosphorylation [21], [38].

miR-146a transient overexpression in epiphyseal cells (low level expression of miR-146a compared to diaphyseal cells under normal culture conditions) for 48 hours resulted in a significant down-regulation of SMAD3 at the mRNA level with reduction of SMAD2 and SMAD3 protein level at 72 hours, evidenced by western bolt analysis. Concomitant with the reduced SMAD2 and SMAD3 levels observed, reduced expression of the chondrogenesis related gene SOX9 and up-regulation of the osteogenesis related gene RUNX2 was observed. These data suggest miR-146a is a negative regulator of chondrogenesis through down-regulation of SMAD2/SMAD3 and may indirectly promote osteogenic differentiation. Furthermore, as over-expression of miR-146a resulted in a significant negative effect on chondrogenesis in culture over a 48–72-hour period, the effect of miR-146a under normal physiological conditions would appear to be significant. To optimize our transfection protocol, miR mimics against GAPDH were used and shown to reduce GAPDH expression by over 80% after 48 hours post transfection. However, miR inhibitors were found to have display a much lower efficacy. Coupled with the high expression of miR-146a in diaphyseal cells, it proved difficult to reproducibly demonstrate the effects of miR-146a inhibitor on human foetal diaphyseal cells.

In the current study, we observed that cells extracted from the epiphyseal and diaphyseal regions of a developing human foetal femur retain their progenitor cell characteristics, evidenced by the expression of CD63, ALCAM and Nucleostemin mRNA, but exhibit different and distinct phenotypes and display discrete affinities in differentiation along the osteogenic and chondrogenic lineages. Foetal epiphyseal cells expressed higher levels of miRs including miR-140 and miR-138 reported to promote chondrogenesis [20]–[22] while diaphyseal cells expressed miRs including miR-210 and miR-93 previously reported to promote osteogenesis [3], [23], [39]. These data suggests the chondrogenic epiphyseal cell population expressed increased levels of miRs associated with chondrogenesis while the osteogenic diaphyseal cells expressed miRs associated with osteogenesis.

**Figure 9:**
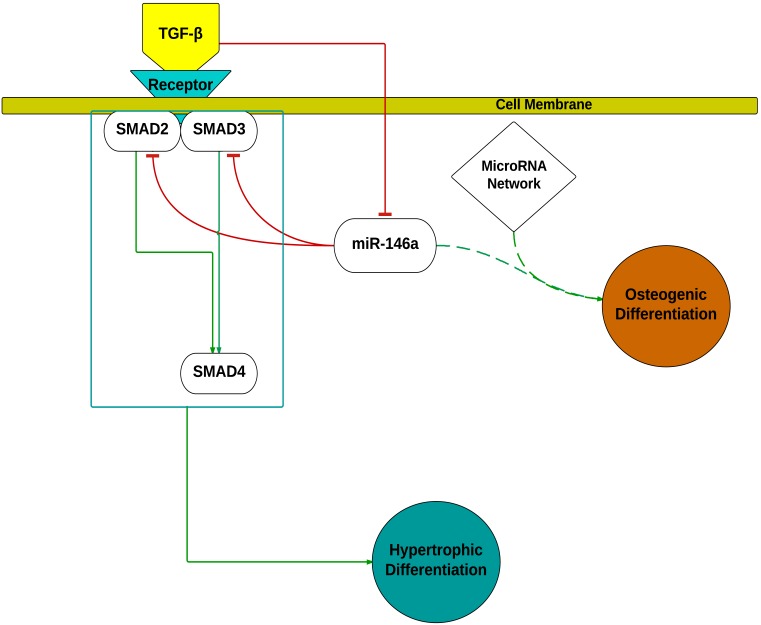
Modulation of skeletal cell differentiation by miR-146a. miR-146a down-regulated SMAD2 and SMAD3 protein levels, resulting in an attenuation of TGF-β signaling following TGF-β3 stimulation. Stimulation by TGF-β3 down-regulated the expression of miR-146a indicating a double negative feedback loop and thus a potential auto-regulatory mechanism. Overexpression of miR-146a under basal condition revealed a modest, but positive effect on osteogenesis related gene expression and is likely part of a miR network involved in promoting osteogenesis. The inhibitory effect of miR-146a on SMAD4 was previously reported by Zhong *et al* [31] and was not reevaluated in this study.

TGF-β signaling is important for skeletogenesis. It is generally accepted that the bone morphogenic proteins (BMPs) and their receptors induce early cartilage formation and stimulate mesenchymal cells to differentiate into osteoblasts whilst TGF-β ligands and their receptors regulate chondrocyte proliferation and differentiation [12], [40]. In the current study, the effect of TGF-β3 stimulation in monolayer culture model were consistent with current literature with TGF-β3 stimulated cells differentiating to give hypertrophic chondrocytes coupled with a significant up-regulation of Type X collagen mRNA expression [21], [24], [40]. TGF-βs signals are known to be transduced to the nuclei by the intracellular mediators, SMADs [41]. To date, eight different SMAD proteins have been identified, classified into three categories based on their functions; the receptor-activated SMADs (SMAD- 1, 2, 3, 5 and 8), common mediator SMAD (SMAD4) and the inhibitory SMADs (SMAD6 and 7) [41]. The receptor-activated SMADs are further divided into two groups based on their attachment to ligand specific receptors; SMAD2 and 3 transduce signal by TGF-βs and activin ligands while SMAD1, 5 and 8 respond to BMPs stimulation [42]. As SMAD2 and SMAD3 are known to transduce TGF-β ligand signals [43], the effect of miR-146a on the TGF-β pathway was examined through analysis of the effects of miR-146a transient overexpression on cells stimulated by TGF-β3. In the absence of miR-146a overexpression, TGF-β3 was observed to up-regulate the expression of Type X collagen 2000-fold. However, following transfection with miR-146a mimic, TGF-β3 induced up-regulation of collagen X was reduced by over 60%. These results suggest miR-146a attenuates the TGF-β3 ligand signal, and possibly activin signals, through down-regulation of SMAD2 and SMAD3 protein. Interestingly, when cells were stimulated with TGF-β3, a reduction of miR-146a expression was observed. The current data indicate the presence of a negative feedback mechanism between TGF-β3 stimulation and miR-146a expression advocating the presence of an auto-regulatory mechanism (Figure 9). Thus miR-146a may reduce TGF-β signaling and in turn TGF-β3 stimulation may suppress miR-146a expression. A similar auto-regulatory feedback mechanism has also been described for the regulation of miR-93 and downstream Osterix expression during osteoblast mineralization [3] as well as for miR-140 on TGF-β signaling through SMAD3 suppression [44].

Various reports have already demonstrated the importance of miRs during SSC differentiation; miR-140, a cartilage specific miR [19], was reported as a positive effector of chondrogenesis through PDGF signaling in zebrafish [45]. In addition, miR-140 has been linked to the regulation of SMAD3 dependent TGFβ pathway through down regulation of SMAD3 protein levels and thus to play a role in chondrocyte development [44]. miR-138 has been shown to inhibit osteogenic differentiation in telomerase immortalized bone marrow derived hMSC through down-regulation of FAK and subsequently down regulation of the FAK downstream targets RUNX2 and Osterix [21]. These studies demonstrate various miRs may work in concert to regulate the complex mechanisms underlying SSC differentiation. Together with the distinct expression patterns observed in epiphyseal and diaphyseal cell populations, the data in this study suggests, by extracting cells from distinct regions of the human foetal femur, a more homogenous skeletal stem cell population can be isolated, providing an innovative approach for identification of novo miRs involved in skeletal stem cell differentiation and skeletogenesis.

## Conclusion

This report has further characterized foetal derived SSC found in foetal femur by assessing differentiation related gene expression and miR expression profile in epiphyseal and diaphyseal cells. Using miR array, miR assay and transient over-expression analysis, we have identified miR-146a as an important regulator of TGF-β signaling during chondrocyte development and by extension, foetal skeletogenesis, through regulation of SMAD2 and SMAD3 protein translation. Further functional analysis to define the precise role of miR-146a and other miR targets identified in the epiphyseal and diaphyseal cell populations could provide a novel strategy to manipulate SCC differentiation in regenerative medicine applications.
